# Understanding the Causes of Keel Bone Damage and Its Effects on the Welfare of Laying Hens

**DOI:** 10.3390/ani14243655

**Published:** 2024-12-18

**Authors:** Xin Li, Xia Cai, Xiaoliang Wang, Lihui Zhu, Huaxiang Yan, Junfeng Yao, Changsuo Yang

**Affiliations:** 1 Institute of Animal Husbandry and Veterinary Science, Shanghai Academy of Agricultural Sciences, Shanghai 201106, China; lixinxinli_sadu@outlook.com (X.L.); acandaf@163.com (X.C.); wxlyjf@163.com (X.W.); zlh309@163.com (L.Z.); 15921982837@139.com (H.Y.); 2 National Poultry Engineering Technology Research Center, Shanghai 201106, China

**Keywords:** laying hens, keel bone damage, influencing factors, evaluation method

## Abstract

Keel bone damage (KBD) is a prominent concern within the realm of the egg-laying industry, exerting substantial impacts on the welfare and productivity of laying hens. This comprehensive review undertakes a detailed exploration of the diverse factors contributing to KBD, such as inadequate calcium sources in the medullary bone, genetic factors, nutritional deficiencies, and physical stressors. The consequences of KBD on production performance, stress and inflammation levels, and the physical and chemical properties of the keel are meticulously examined. Additionally, the review evaluates the existing methods for assessing KBD, including keel curvature scoring, imaging techniques, palpation, biomechanical testing, behavioral observations, and biochemical markers. Finally, management strategies, including nutritional adjustments, genetic selection, and environmental modifications, are proposed to potentially mitigate the prevalence and severity of KBD, thereby aiming to enhance the welfare and productivity of laying hens.

## Simple Summary

Keel bone damage (KBD) in laying hens represents a significant issue with profound implications for both the welfare and productivity of these birds. This review systematically investigates the etiological factors, consequences, and assessment modalities of KBD, and further proposes corresponding management strategies. The occurrence of KBD can be ascribed to multiple factors, including insufficient calcium supply, genetic determinants, and physical stressors, which may give rise to alterations in egg production, stress responses, and inflammatory states. Assessment methods encompass palpation and imaging techniques. Management strategies comprise nutritional optimization, genetic selection, and environmental adaptation. Comprehending and addressing KBD is of substantial importance for the sustainable development of the poultry industry and the safeguarding of laying hen welfare.

## 1. Introduction

Keel bone damage (KBD) in laying hens is a significant welfare issue with implications for productivity and sustainability in the egg-laying industry. This review explores the causes, impacts, and assessment methods of KBD, and proposes strategies for its management and prevention.

In intensive egg production facilities, such as battery farms, cage conventionals compress the living space of laying hens (also referred to as ‘layers’), limiting their free expression of innate behaviors, such as flapping of wings, flying, stretching, and dust bathing. Additionally, other factors, such as earlier onset of lay, indicated by age at first egg, is associated with an increased risk of keel bone damage, possibly due to the immature bone structure being unable to withstand the stresses associated with egg production [1]. It has been reported that osteoporosis is responsible for 20–35% of deaths in laying hens housed in conventional cages [2].

Based on such welfare considerations, the breeding of layers is gradually changing from conventional laminated or stepped cages to enriched cage and non-cage systems with access to larger spaces. The implementation of cage-free systems allows laying hens to engage in more natural behaviors, but, as a result, increases the risk of KBD, which can be painful for the birds [3].

In addition, the breeding selection for precocity and high laying rate of commercial laying hens gives rise to traits of concern. Continuous selection increases the laying load and the metabolic rate of calcium in the body, resulting in increased fragility in, and damage to, the bones of laying hens. Breeding strategies to reduce keel bone damage in laying hens focus on selecting for stronger bone genes, optimizing nutrition and husbandry, improving housing design, and exploring the use of analgesics and gene editing. Despite advancements, the high rate of KBD, affecting up to 97% of hens in certain regions, remains a major welfare concern. The adverse impact of KBD, compounded by factors such as high egg production rates [4,5] and calcium metabolism [6], underscores the urgency for in-depth research to identify its root causes and establish preventive measures. The keel bone plays a crucial role in the expression of important behavioral processes, such as flight, jumping, and breeding, and physiological functions, including respiration and temperature regulation [7]. However, KBD accounts for more than 90% of fractures in laying hens [8], rendering it one of the most common fracture sites in such birds. Thus, KBD of various types, including bone fractures and deviations, have become significant problems in the egg-laying industry [9]. For example, the KBD rates of commercial laying hens during the late laying period in Belgium, The Netherlands and the UK are as high as 97%, 97%, and 86%, respectively [10,11], with levels of 50–80% reported in other countries [9,12,13]. Such a high incidence of KBD is an cause for concern. KBD in chickens cause pain through various mechanisms, as evidenced by behavioral and physiological changes, and this pain negatively affects their welfare [14]. The increasing prevalence of keel bone damage in laying hens has garnered significant attention from researchers in the field of animal welfare. Recognizing the potential impact of this condition on the overall well-being and productivity of egg-laying birds, scholars have dedicated considerable effort to investigating the causes, consequences, and potential mitigation strategies associated with keel bone damage. Thus, we summarize here the causes, progress in understanding, and main methods of evaluating KBD, and provide novel perspectives for further research into KBD in the egg production industry. These KBD, whether slight or severe, represent a chronic welfare problem in laying hens.

## 2. Characteristics of Keel Bone Damage

### 2.1. Definition and Types of Keel Bone Damage

KBD refers to fractures and deviations of the keel bone in birds [7]. A keel fracture is a fracture that results in displacement, cracking, sharp edges, and healing scars in the keel [15]. Such fractures are characterized by a sharply curved, shear, and/or broken portion of the keel [15]. Fracture severity is influenced by tissue properties, such as the degree of mineralization, mineral composition, crystallinity, collagen properties and bone cell viability, and by structural properties, such as cortical thickness, porosity, trabecular thickness, and connectivity [16]. However, attempts to identify the exact cause and predisposing risk factors for the fractures to develop may have been hampered by the lack of a clear distinction between keel bone fractures and keel bone deviations, with overlapping nomenclature (e.g., keel bone deformities, KBD, keel bone disorders, crooked keels, etc.) [17].

Keel bones deviations are defined as ‘bone[s] with an abnormally shaped structure that has not resulted from a fracture but contains section(s) that vary from a theoretically perfect 2-dimensional straight plane in either the transverse or sagittal planes’ [7,18]. In addition, up to 82% indentation along the ventral surface can also classify a keel as being ‘curved’ [7]. Studies reported that 6–59% of laying hens aged 60–62 weeks have keel bone deviations [19,20]. Animal behavior experiments showed that layers without KBD took less time to complete a runway test, spent less time flying from the ground to perches of different heights and from these perches to the ground, and spent less time sleeping on the floor compared with layers with keel damage [3]. The authors speculated that the damage to the keel caused pain and affected the normal behavior of the laying hens.

### 2.2. Impact on Production Performance

Keel bone damage (KBD) in laying hens not only affects their overall well-being and productivity but also presents a multifaceted challenge with significant implications for egg production systems. In addition, KBD reduces the laying potential of hens [21,22] and, thus, is negatively correlated with egg production [3,10,20]. It has been hypothesized that the difference in egg production between layers without keel fractures and those with keel fractures might be due to pain or stress affecting hormones associated with ovulation or reducing the granular cell response to luteinizing hormone [23,24]. Eggshell thickness was reported to be similar between layers with and without a keel bone fracture, but the eggs of those without a fracture were found to be heavier [14]. Heavier eggs usually have a larger surface area [25] and, thus, require more calcium to form the shell. Where a keel bone fracture occurs, calcium can be used for bone healing and callus formation rather than for eggshell formation, leading to smaller, poorer eggshell-quality eggs [26]. Such conclusions are supported by studies that reported a positive correlation between good bone strength and good eggshell quality [27,28].

### 2.3. Effects on Stress and Inflammation

In birds, the keel is a long structural bone extending along the central axis of the sternum and perpendicular to the ribs, providing anchoring sites for the breast muscles. As a result of internal air-filled spaces, the keel also provides buoyancy to aid flight [29], as well as having an important supporting role in the physiological behaviors of birds, such as flapping wings and breathing [30]. Given that birds lack a muscular diaphragm, they rely on the movement of their keel and ribs to drive inhalation and exhalation [31]. If the keel is severely damaged, causing pain or limiting its movement, its ability to aid breathing may be reduced, in turn affecting the behavior as well as metabolic or thermoregulatory abilities of the bird. When the behavior of laying hens 10 days before and 10 days after a keel bone fracture were compared, it was found that the hens remained in the nest for longer during the laying period after compared with before the fracture [32]. Layers with no fractures spent more time roosting on the floor, whereas those with minor fractures or severe keel damage roosted less on the floor and spent more time roosting on perches [23]. In addition, KBD has a negative effect on the emotional behavior of laying hens. In mammals, stress-induced reductions in mature hippocampal neurogenesis can lead to depression-like states, and similar reductions in laying hens may impair cognitive function, alter social behavior, and increase stress susceptibility [33,34].

Previous studies found that KBD could cause psychological stress measured by fear-related tests [35] and physiological stress measured by blood corticosterone [36]. Correspondingly, serum levels of interleukin-1β (IL-1β) and IL-6 were increased, and peripheral serotonin concentrations were decreased [37], indicating that keel bone fractures induce fear (physiological reactions akin to fear responses) in laying hens to a certain extent.

Fractures can cause physiological stress, which increases stress hormone levels and pain, as well as inflammatory responses [38]. The physiological inflammatory response that occurs after fracture is considered to be the protective response of tissues to harmful stimuli and injuries, and is committed to the clearance of harmful stimuli and initiation of bone healing. The acute inflammatory response after injury peaks within the first 24–48 h after injury and usually last for ~7 days [39]. As the fracture heals, acute inflammation is replaced by chronic inflammation, which is a state of simultaneous acute inflammation, fibrosis, and repair, which can last for weeks, months, or even years. Expression of TNF-α, NF-κB p65, iNOS, PTGEs, COX-2, and IL-1β increased in the brain, liver, and chest muscles of laying hens with keel fracture [35]. This suggests that keel bone fracture causes an inflammatory response in laying hens, leading to the production of numerous inflammatory cytokines at the fracture site.

### 2.4. Changes in the Physical and Chemical Properties of the Keel

A keel bone fracture is identified as having occurred if, on palpation, a bony callus is present on the ventral or lateral surfaces of keel bone, which is a result of the regenerative healing process following a fracture [40]. Bone strength refers to the toughness of the bone or its ability to withstand stress, and is the sum of the forces and movements applied to the bone. In precise terms, it is the load or stress that is normalized to represent the force applied per square area at the time of fracture. In general, bone strength is related to its physical (shape, size, and mass), structural (collagen fiber orientation), and material (matrix molecules) properties. Keels deformed by deviations or fractures will have different strengths compared with healthy keels; although material properties, such as mineral and organic substrates, will be similar, changes in matrix properties, such as low calcification resulting from osteomalacia or excessive hydroxylation of collagen, alter bone strength. Bone mineral density reflects the amount of bone mineral per volume of bone, which includes both organic and mineral mass, thus it can reflect the health status of bone. Low bone mineral density is associated with a higher risk of fracture. Hocking et al. compared commercial layer varieties with high egg production performance with traditional layer varieties with significantly lower egg production performance, and found that the latter had higher X-ray photographic density and higher fracture strength [41]. The bone density of laying hens that do not lay eggs was found to be higher than that of laying hens, which might explain the higher incidence of fractures in laying hens. In addition, increases in keel bone density reduced the likelihood of experimental keel fractures [41].

## 3. Possible Influences on KBD

### 3.1. Insufficient Calcium Source in Medullary Bone

#### 3.1.1. Types and Functions of Bones

**Figure 1:**
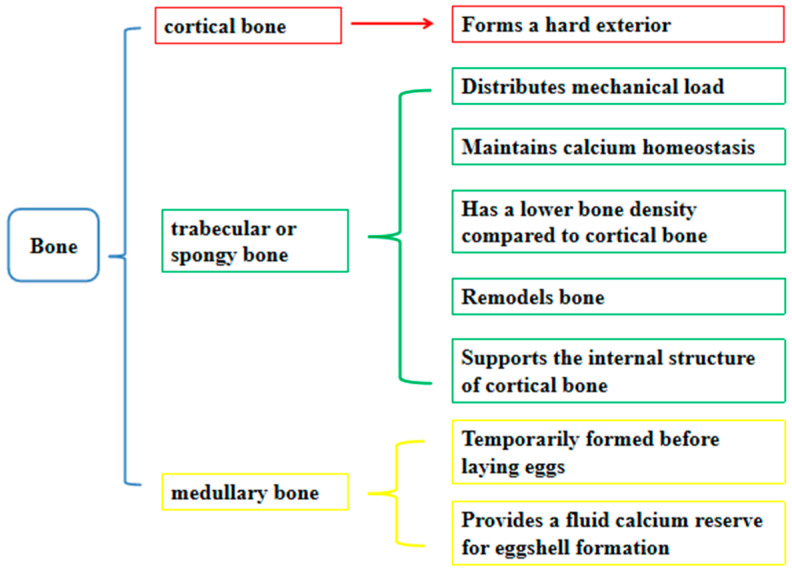
Types and functions of bones. The chart uses different colors to distinguish and emphasize different content.

Bone can be divided into cortical bone, trabecular or spongy bone, and medullary bone (see Figure 1). Cortical bone forms the hard exterior, while trabecular bone helps distribute the mechanical load and maintain calcium homeostasis. Medullary bone is temporarily formed before laying to provide a fluid calcium reserve for eggshell formation. Loss of cortical bone can put the keel at risk of deviation or fracture. By contrast, trabecular bone has a lower bone density compared with cortical bone, and its main functions include distributing the mechanical load, remodeling bone, and maintaining systemic calcium homeostasis, which helps to support the internal structure of cortical bone. Keel bone deviation is considered to be a breakdown of the bone surface around the keel and, therefore, might not be a direct result of fracture or impact injury [42]. Keel bone deviation might occur over a period of time as bone remodeling responds to conventional load stress.

#### 3.1.2. Calcium Requirements and Metabolism

Nearly 20–40% of the calcium required for eggshell formation is provided by bones [43] in the form of hydroxyapatite, which provides stiffness and compressive shear strength to bones by binding neighboring collagen fibers. The arrangement of collagen molecules causes them to overlap, creating repeated periodic pore region fragments in collagen fibers where hydroxyapatite crystals are deposited and calcification occurs. Calcium and phosphorus comprise 95% of the bone mineral matrix. Laying hens are more prone to osteoporosis due to the high calcium demand, and a negative calcium balance can lead to bone damage. Before laying eggs, birds temporarily form medullary bone tissue to act as a fluid calcium reserve to aid eggshell formation, but this reserve is reabsorbed after laying is complete. The skeleton of hens is fully formed and has stopped growing at the point at which they are able to lay eggs. However, bone density and content, as well as the proportion of cortical, trabecular, and medullary bone, can vary dramatically. Although medullary bone is the most unstable bone type, cortical bone and trabecular bone are also mobilized as calcium sources if laying hens are deficient in calcium. During laying, hens gain a net amount of bone mass because of medullary bone formation and structural bone loss. In fact, the volume of trabecular bone in laying hens decreases by half between 16 and 31 weeks of age [44]. Compared with cortical bone, trabecular bone has a lower degree of calcification, which is related to its bone remodeling and metabolic function [45]. Medullary bone is lower in collagen compared with cortical or trabecular bone, but higher in minerals, proteoglycans, and carbohydrates [46]. Although medullary bone lacks substantial strength, it can affect the mechanical strength of cortical bone [47,48]. In female birds, medullary bone with a higher rate of remodeling develops on the inner surface of the long bone as the follicles mature, providing calcium needed for eggshell formation [49].

#### 3.1.3. Bone Maturation and Keel Ossification

**Figure 2:**
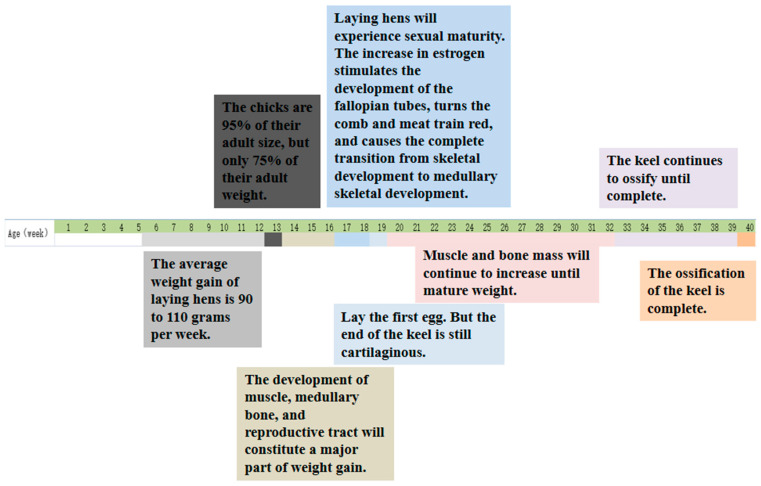
Timeline of keel bone maturation [1,7,49,50]. In the figure, different colors are used to distinguish various stages and events in the development of laying hens and keels.

Bone maturation involves complex molecular and biochemical changes to achieve optimal mechanical strength. In laying hens, keel ossification is complete at 40 weeks of age. Bone maturation refers to completion of the basic structural development and mineralization of bone through complex molecular and biochemical changes to achieve optimal mechanical strength. These changes occur in collagen fiber diameter, cross-linking content, and deposition of more multilayered bone. The amount of collagen cross-linking is an indicator of bone maturity in mammals [50]. As birds age, cortical and trabecular bones become thinner, and structural bone integrity usually decreases, whereas the medullary bone content increases. Birds that produce good-quality eggshells do so through the loss of cortical bone to release calcium, but this occurs at the expense of medullary bone structures and a higher risk of keel deviation or fracture. The incidence of fracture often increases sharply from the beginning of laying to the peak laying period (~25–35 weeks of age), before slowing down after 49 weeks, although the incidence of keel fracture can reach 100% by the end of laying [51]. The increase in estrogen stimulates the development of the fallopian tubes, turns the comb and wattle red, and causes the transition from skeletal development to medullary skeletal development. The first egg is laid at around 19 weeks of age, when the keel is still cartilaginous to a certain extent (Figure 2). Hens with broken keel bones at depopulation had laid their first egg earlier than hens with intact keel bones [1,50]. This might be because a large amount of calcium is required for eggshell formation to begin egg laying, and for hens with high egg production, the cartilaginous keel might not receive enough calcium at the beginning of egg laying to achieve normal ossification. Subsequently, muscle and bone mass continue to increase until the hens reach their mature weight, at ~32 weeks of age. Researches show that only 0.8% of 15-week-old pullets had deformed keels, and keel deformity was not evident in pullets prior to lay, but incidence of deformities appeared to increase during the laying period [7]. In the event that the uniformity of flock weight is suboptimal prior to the laying of the first egg, the peak of egg production will be affected. In such a flock, the chickens with lighter weights will not be put into production promptly upon reaching or exceeding the ideal weight, and these birds exhibit fewer cases of Keel Bone Damage (KBD) as they commence laying at a later stage. There is a view that the rapid growth of poultry weight outpaces the development and maturity of their bones, resulting in excessive body load and making bones easily deformed and fragile. These results suggest that reaching bone maturity or the maximum physical and functional potential of bones takes longer than the growth process itself. Bone loss in laying hens results from persistent dynamic changes in structural bone that lead to bone fragility and fracture susceptibility. The degree of medullary bone mineralization at sexual maturity is positively correlated with bone quality at the late laying stage.

#### 3.1.4. Effects of Calcium Deficiency

Insufficient calcium can lead to keel deviation and fracture [6]. During peak laying, hens need calcium for eggshells and a deficiency (due to diet or excessive bone mobilization) causes decreased bone density, structural changes, and affects medullary bone, making bones fragile. Long-term deficiency can result in a 62% incidence of KBD at depopulation [51]. Hens with early egg-laying may have insufficient calcium for normal keel ossification, increasing the risk of keel damage. More fractures occur during the highest laying rate, and hens with deviated keels are more likely to develop fractures later. During laying, only medullary bone formation occurs while structural bone resorption continues; structural bone reforms only when hens are removed from laying cages [52,53]. Insufficient bone mass can lead to keel fractures [54]. Laying hens are prone to keel curvature and fracture before 40 weeks of age, and after 40 weeks of age, keel injuries may mainly be fractures due to complete ossification. Some research shows fractures increase with age, peaking at 50 weeks and then leveling off [12,55,56]. Keel deviation and fracture mainly occur during 15–50 weeks of age, with fracture being the main damage after 50 weeks and early KBD incidence (before 15 weeks) being less than 0.8% [52,53]. The interaction between calcium deficiency, estrogen, and bone metabolism stages is pivotal in understanding keel bone damage. Estrogen stimulates osteoblast function and inhibits osteoclast function [53,57], but the high demand for calcium during peak laying leads to increased osteoclastic activity and bone resorption. This resorption is not confined to medullary bone, which explains the susceptibility of laying hens to keel bone damage [58]. The dynamic balance between bone resorption and formation is crucial for maintaining bone quality, and disruptions in this balance due to calcium deficiency can lead to osteoporosis and increased fragility of the keel bone [59].

### 3.2. Genetics

#### 3.2.1. Heritability of KBD

Researches show that KBD is a heritable trait with medium heritability (the heritability of 30- and 46-week-old hens was found to be 0.26 and 0.24, respectively), indicating that KBD is affected by genetic factors [60]. Genetic selection could be an effective method for changing the KBD level in the chickens. Bone density also has been found to be hereditary in humans and other species [61]. Some genes are responsible for regulating calcium metabolism and bone development. Variations in these genes can affect the efficiency of calcium absorption, transport, and deposition in bones. For example, certain genetic mutations may lead to a decreased ability to absorb calcium from the diet, even when calcium supply is adequate [62]. Genetic factors can also influence the structure and quality of bones. Genes involved in collagen synthesis and bone cell regulation can impact the strength and integrity of the skeletal structure [63]. If these genes are mutated, bones may be more prone to damage and fractures.

#### 3.2.2. Genetic Mutations and Bone Health

Mutations in collagen gene can affect bone collagen synthesis, fiber formation, or its post-translational modification, thereby altering matrix chemistry and mineralization, thus, affecting bone strength [64]. Tibial dyschondroplasia (TD) is hereditary in poultry [65]. It affects the growth plate of the proximal tibia. This disease can indirectly contribute to osteoporosis during early growth by altering mechanical structure, disrupting hormone regulation, and changing nutrient allocation [66]. Genetic mutations can directly lead to the occurrence of TD. Mutations in specific genes involved in bone and cartilage development can disrupt the normal processes of growth and maturation. For instance, mutations in genes that regulate chondrocyte proliferation and differentiation can cause abnormal cartilage formation at the growth plate of the tibia, a hallmark of TD [67]. These mutations may affect the signaling pathways that control the balance between cartilage formation and its conversion to bone. Genetic mutations can also influence the metabolism of nutrients and growth factors that are essential for proper bone and cartilage development. For example, mutations in genes related to vitamin D metabolism or calcium homeostasis can disrupt the mineralization process and contribute to the development of TD [68]. Moreover, multiple genes may interact with each other and with environmental factors to increase the susceptibility to TD. Some genetic backgrounds may make poultry more vulnerable to certain environmental stressors, such as nutritional imbalances or infectious agents, which can trigger the manifestation of TD.

#### 3.2.3. Breeding Lines and KBD

Studies have found that some local varieties without high intensity commercial selection and breeds may have a lower risk of KBD compared to highly productive egg lines. There was also a difference in the incidence of keel injury between white and brown shell-laying hens [56]. Genetic predisposition within different breeding lines can influence the likelihood of KBD. For example, Growth traits selected in breeding lines, such as rapid growth and high egg production, can also impact bone health [69,70]. Rapidly growing birds may lack proper bone development time, while high egg production can deplete calcium reserves. Additionally, body size and conformation play a role, as larger birds and certain conformations can put more stress on the keel bone [7]. Understanding this relationship has implications for breeding and management. Selective breeding for traits promoting bone health and tailoring nutrition and housing conditions can help reduce KBD incidence and improve poultry welfare.

#### 3.2.4. Candidate Genes

The selection elimination analysis based on the differences in keel phenotypes between White Leghorn laying hens identified 10 important candidate genes that have strong selection signals and are related to bones. These genes are *ACP5*, *WNT1*, *NFIX*, *CNN1*, *CALR*, *FKBP11*, *TRAPPC5*, *MAP2K7*, *RELA*, *ENSGALG00000047166* [60]. Furthermore, a genome-wide association study of bone quality in 1534 hens using a 600K chicken genotyping array identified three candidate genes that may be associated with osteoporosis in laying hens, namely *RANKL*, *ADAMTS* and *SOST* [43].

### 3.3. Nutrition, Collagen and Bone Health of Laying Hens

A mildly to moderately deficient diet may not cause bone problems until after the peak in laying, but for underweight laying hens, nutrient deficiencies can quickly affect egg production [71]. For underweight laying hens, nutrient deficiencies affect egg production more quickly [72]. Mild to moderate nutrient deficiencies usually lead first to bone and/or shell quality issues, followed by production issues, whereas severe nutritional deficiencies can lead to significant and rapid declines in yields [71].

#### 3.3.1. Nutrients and Bone Health

Calcium and phosphorus are crucial for bone strength, and their imbalance can affect bone integrity and strength [62]. High levels of phytase and cellulose fiber in the diet can interfere with calcium absorption, while moderate fluoride and boron can have positive effects on bone density and strength [73]. Vitamin D is important for calcium absorption and bone metabolism, and vitamins B6, C, and K are involved in the synthesis of matrix components and collagen crosslinks [68]. Excessive protein intake can create a negative calcium balance, and a diet high in saturated fat can have an adverse effect on bone mineralization [74]. Laying hens are more likely to develop osteoporosis when laying eggs, because of the negative calcium balance caused by the high demand for calcium during eggshell formation [42,52]. During the day, egg shell calcium comes from intestinal absorption and medullary bone breakdown; At night, when the light is off and the hens stop eating, medullary bone provides more calcium for eggshell formation [75]. In general, the eggshell formation period is mainly at night, and the body needs to provide 2.0–2.5 g calcium to synthesize an eggshell [76]. In the process of stopping light at night, 25% to 40% of the calcium in the eggshell is obtained through the bone melting of medullary bone [77]. Negative correlations have been reported between egg production, eggshell thickness, and bone fracture strength [76], and an imbalance in phosphorus metabolism can also affect bone integrity and strength [78]. Moderate fluoride can increase the bone density of laying hens, whereas boron can improve bone strength [47]. Copper affects the formation of collagen crosslinks and, thus, bone mineralization [79]. Trace amounts of aluminum are associated with osteomalacia and osteoporosis, while excess aluminum can cause growth inhibition and reduce bone strength [80]. Vitamin D is a hormone involved in the absorption of calcium in the intestine and has an important regulatory role in bone metabolism and bone strength. Low calcium stimulates parathyroid hormone secretion and vitamin D synthesis, which in turn activates the release of bone minerals. Vitamin D is a key factor in promoting the activity of osteoblasts in cortical and medullary bone [81]. In addition, vitamin D is involved in medullary bone formation. In osteoblasts, estrogen can increase the expression of vitamin D receptor, stimulate the secretion of osteocalcin, and co-operate with vitamin D to promote medullary bone formation. Vitamins B6, C, and K are involved in the synthesis of matrix components, such as collagen and osteocalcin, and the formation of collagen crosslinks [82]. Protein and carbohydrates as energy sources are important for bone health, but excessive protein intake can create a negative calcium balance, hindering bone growth [83]. A diet high in saturated fat can have an adverse effect on bone mineralization, whereas a low-fat diet can increase trabecular bone strength and bone mineral content [84].

#### 3.3.2. Collagen and Bone Health

Collagen, a triple helix fibrous protein, is the main scaffold for the formation of bone tissue, enhancing the tensile strength of bone and providing directional support for bone mineralization [85]. In addition, collagen molecules undergo fiber formation and other post-translational modification processes, such as hydroxylation and intermolecular crosslinking, and ‘pyrrole’ crosslinking contributes to the biomechanical strength of bone [86]. In addition, non-collagen proteins contribute to matrix stabilization, calcification, and other metabolic regulatory activities of bone [87]. There was a high correlation between changes in bone collagen crosslinking content and bone strength between young and mature birds, but only a 5–10% difference in ash content and density [88].

### 3.4. Physical Factors

#### 3.4.1. Collisions in Loose-Housed Systems

In loose-housed systems, collisions play a significant role in the occurrence of KBD in laying hens. One physical reason suggested for KBD is that loose-housed (i.e., non-cage) systems collisions contribute to an increased risk [59,89]. In such non-cage environments, hens have more freedom of movement, which leads to various types of collisions such as with housing structures, perches, and other hens. The design of the housing system, including factors like layout and spacing of elements, along with the hens’ natural behaviors, contribute to these collisions. The keel of poultry is usually the first point of contact during a collision, making it easily damaged [90]. When laying hens roost, most of the weight of the body is supported by the keel. As a result of these collisions, various health issues can arise. Bone injuries like keel bone fractures, deviations, hematomas, and wounds are common [10]. Additionally, foot health problems such as dermatitis and hyperkeratosis may also occur [91]. Several factors influence the collision rates. Housing system design aspects like the type of aviary, flooring material, corridor width, and nest box entrance are crucial [92]. Different hybrids of laying hens have varying susceptibilities to collisions and resulting injuries due to genetic factors. Management practices, including providing a clean and dry environment, adequate lighting, and proper stocking density, also play a part in reducing collision risks and thereby enhancing the welfare of laying hens in loose-housed systems [93].

#### 3.4.2. Cage Conditions and KBD

Although caged laying hens may have a higher percentage of normal keels overall compared to loose-housed hens, a high prevalence of keel bone fractures and deviations has also been found in traditional cages [58]. Several factors contribute to this issue. Cage design and material are crucial aspects. Narrow cages restrict the movement of hens and increase the risk of friction between the keel and cage surfaces, potentially leading to damage [94]. Studies have shown that in caged laying hens (three-layer H-shaped cages), the KBD of the bottom cage showed more severe, indicating that the height of the cage is an important factor affecting the level of KBD [21,60]. Moreover, certain hard materials can cause collisions with the keel, resulting in subsequent trauma. Stocking density also plays a vital role. Excessive numbers of hens per cage lead to overcrowding and increased collisions, thereby raising the likelihood of keel damage [92,95,96]. Prolonged caging duration is positively associated with the risk of KBD. Reduced activity due to caging leads to muscle atrophy, decreased bone density, and impaired balance, all of which heighten the risk of keel damage [2]. Additionally, body weight [97] and perch design are factors that influence KBD risk in both cage and non-cage systems.

#### 3.4.3. Pressure on the Keel

Long-term pressure on the keel during roosting is a risk factor for KBD, as the pressure load on the keel is significant. This is primarily because the pressure load on the keel during this period is substantial. In fact, Laying hens mainly support their body weight on the keel during roosting, and the pressure load on the keel is five times that on a single foot pad [20]. Thus, the design of perches could influence the risk of keel deviation in chickens. For instance, the shape, size, and material of the perch can all impact the way a hen perches and the distribution of pressure on the keel. In addition to the design, the placement of perches within the housing environment also matters. Research has indicated that the increased severity of keel deviation during feeding is associated with perch use [98]. Specifically, research showing that the presence of perches led to the buckling and even fracture of the keels of the hens [29]. This may be due to the hens’ movements and postures while using the perches during feeding activities, which can cause abnormal stress on the keel [99]. Furthermore, the type of structure provided in front of the nest box can have an impact on keel health [91]. Providing a perch in front of the nest box instead of a platform could reduce the number of collisions or scraping of the keel skin, thus reducing the amount of damage sustained. This is because a perch may offer a more natural and stable resting place for the hens as they approach or leave the nest box, thus reducing the amount of damage sustained by the keel. In addition to the factors related to perches, the chest muscle mass of the hens also has an influence on keel fracture susceptibility [100]. Lower chest muscle mass can render the keel more prone to fracture as there is less muscle support to absorb and distribute forces. Conversely, higher chest muscle mass can provide some protection to the keel by acting as a buffer and sharing the load during various activities [16].

**Figure 3:**
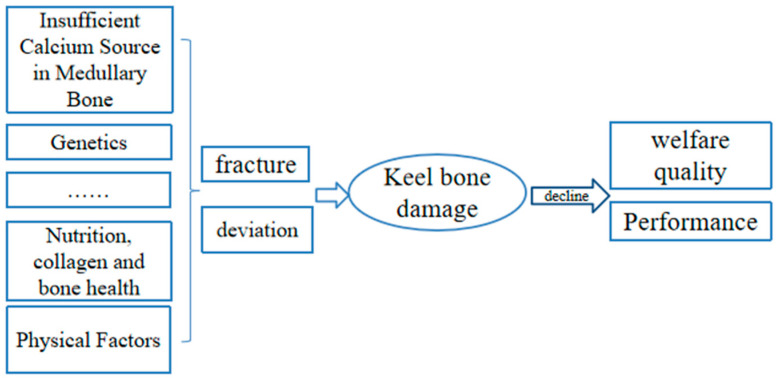
Characteristics and possible causes of keel bone damage in laying hens.

In summary, keel bone damage in laying hens is caused by multiple factors (see Figure 3), and it has serious adverse effects on the welfare and production performance of laying hens. In the process of laying hen breeding, attention should be paid to preventing keel bone damage from aspects such as calcium supplementation, genetic screening, environmental improvement, and nutritional balance.

## 4. Assessment Methods

At present, there is no standardized test method for evaluating keel health. Based on previous studies, the main methods of evaluating keel injury included palpation, anatomy, radiography, and computed tomography (CT) scanning, which differed in terms of their reliability, sensitivity, and accuracy. While we believe that palpation will continue to be the chief means of assessing KBD due to its low cost, ease of adoption, and the method being well-validated, alternative methods should be considered as they can offer additional insight that is not possible by palpation alone [32]. New, non-invasive, technologies need to be developed that identify chickens with keel fractures without handling them. The possibility exists that handling itself could contribute to keel bone fractures, especially in aging laying hens as they have to be captured, assessed, and then released during the evaluation process [101].

### 4.1. Keel Curvature Score

The curvature of a keel can be rated based on feeling and observations. The curvature is scored based on a four-point scale according to the Hy-Line method: normal (#1), mild (#2), moderate (#3), or severe (#4). In normal (#1), the keel appears straight with no obvious bending or deformation. When touched by hand, the surface of the keel is smooth and continuous, and the entire structure maintains a uniform and straight linear form from the starting end to the end of the keel. In mild (#2), there are slight signs of bending in the keel. By careful touch, one can feel a slight deviation from the straight line state of the keel line, but this deviation is small and does not affect the overall appearance and normal activities of the chicken. In moderate (#3), the keel shows a relatively obvious bend. When touched, the curvature of the bend can be clearly felt, and the bent part occupies a certain proportion of the keel. In severe (#4), the keel is severely bent with a large degree of curvature and an obviously irregular shape. When touched, a strong deformation of the keel can be felt, and there may be multiple bending points or twisted parts. The higher the score, the greater the curvature of the keel (https://www.hyline.com/Upload/Resources/TU%20SKELETON%20ENG.pdf, accessed on 1 September 2024). In general, the ideal situation is that >90% of keels are scored as #1 or #2. If >10% of keels score as #3 or #4, or there is an increase in the number of birds per week with such scores, it may indicate a problem with the flock’s health or management. This could potentially be related to issues such as nutritional deficiencies, disease, or improper housing conditions that may be affecting the skeletal integrity of the laying hens. Regular monitoring of keel curvature, along with other aspects of skeletal health, is important for maintaining the productivity and welfare of the flock.

### 4.2. Imaging Techniques

Detailed differences in keel integrity, including fractures, can be obtained using imaging techniques. Micro-Computed Tomography (Micro-CT: Micro-CT is a high-resolution imaging technique that uses X-rays to create detailed cross-sectional images of small objects or structures. It can provide a three-dimensional view of the internal structure of a sample with a very fine resolution, often down to the micrometer scale. This allows for the visualization and analysis of complex internal features such as the microstructure of bones, including cortical, trabecular, and medullary bones, without the need to physically dissect the sample.) provides a detailed structure of the inside of the bone without destroying it. It can also be used to reconstruct 3D images that reflect the internal structure of bone [102] and can provide details of the internal structure of cortical, trabecular, and medullary bones that are not possible with other techniques, such as dual-energy X-ray absorptiometry and Quantitative Computed Tomography (quantitative CT: Quantitative CT is a specialized form of computed tomography that focuses on quantitatively measuring the density of a specific material within a scanned object, typically bone mineral density (BMD). It provides numerical values that represent the amount of a particular substance (e.g., calcium in bone) per unit volume. This quantitative information is useful for assessing the health and quality of bones, especially in the context of diagnosing and monitoring conditions such as osteoporosis). Mimics software (Materialise NV, Leuven, Belgium) can be used to convert Computed Tomography (CT: CT is a medical imaging modality that uses X-ray technology to produce cross-sectional images of the body. By rotating an X-ray source and detector around the patient or object being scanned, a series of two-dimensional images are acquired from different angles. These images are then processed using computer algorithms to reconstruct a three-dimensional representation of the internal structures. CT can be used to visualize a wide range of tissues and organs, providing detailed information about their shape, size, and density for diagnostic purposes.) scans into 3D images. However, techniques such as radiography and CT scanning require specialized equipment and grader training and are not suitable for use on general farms. Although imaging techniques can better estimate the true incidence of keel fractures, correlations between some fracture features must be considered during their use and evaluation. At the same time, we should also note that Micro-CT technology encounters several challenges when applied to laying hen bone analysis [103]. Firstly, there are limitations regarding sample size. The relatively small scanning chamber restricts the accommodation of larger laying hen bones, thereby constraining the scope of samples that can be effectively scanned. Secondly, the scanning process presents issues. For larger laying hen bones, the scanning time tends to be longer. This extended duration not only heightens the risk of sample dehydration during scanning, demanding meticulous attention to prevention, but also augments the probabilities of various problems. These include sample movement, thermal drift, misalignment, and beam hardening, all of which can have a detrimental impact on the scanning results and subsequent analyses.

The X-ray absorption capacity of various tissues differs; bone has a relatively high calcium concentration, which can effectively absorb radiation, resulting in an image that can be used to assess for fractures. One of the advantages of using radiography is that recent fractures and fractures involving the back of the keel can be identified, which cannot be achieved by palpation alone [102,104]. However, this technique can only produce 2D images and, in addition, repeated exposure to X-rays can be harmful to the health of both the birds and operators [105].

Ultrasonic bone densitometers are used to measure the sound of speed (SOS) and broadband ultrasound attenuation (BUA) of bone through water or a coupling agent. BUA, Bone Quality Index (BQI), and other parameters related to bone quality, can be calculated to reflect the bone mass value, aiding assessment of the bone condition of laying hens [106]. The basic principle of this approach involves transmitting ultrasonic waves from one side of the bone to the other side, and then measuring the resulting SOS and BUA, which enable calculation of bone density and bone strength, respectively.

### 4.3. Palpation

Keel palpation was developed by Wilkins et al. [8] and verified for accuracy and repeatability by Petrik et al. [55]. The approach involves running two fingers down the side of the keel to feel for calcium deposits or other deformities that indicate previous damage. In addition, attention should be paid to the proximate end of the keel. Skin hematoma and skin wounds can also be evaluated in the keel and the surrounding tissue. For example, skin around the keel might exhibit an inflammatory response [107], which can be visually scored based on a hematoma score. The skin on the keel might also show scabs from a previous sustained wound and, thus, the presence of a wound scab is marked as absent or present. Fracture scores (without or with fracture) can be assigned based on assessment of the medial segment of the keel and at the distal end of the keel (last 1 cm) by palpation. For the total keel score (TKS), scores for hematoma, keel wounds, medial keel fractures, coccygeal fractures, and curvature are added to form a non-isometric score. However, the accuracy of palpation in detecting fractures is limited because this method relies on the rater’s tactile perception of old fractures, and fresh or subtle fractures and fractures on the dorsal side of the keel cannot be detected by palpation alone. In addition, fractures in the distal third of the keel are particularly difficult to detect using palpation, despite the fact that radiographs revealed that 62% of fractures occur in this area. There are different ways to record palpation results. Some methods, like Casey-Trott’s SKAP method, simply record the presence or absence of certain conditions. Others use a wider range of scores, such as 0, 1, 2 or 0, 1, 2, 3. This variety in recording methods poses a problem as it becomes difficult to compare findings across different studies. Without a standardize approach, it is challenging to draw accurate conclusions about the prevalence and nature of keel damage based on palpation data. Therefore, while palpation can provide some initial information, it is not a reliable sole method for evaluating keel fractures, especially in the dorsal and distal areas. To enhance its accuracy and dependability, personnel should be trained in proper palpation fracture assessment techniques.

### 4.4. Biomechanical Testing

Biomechanical testing of keel bones in laying hens is essential for understanding how structural stressors impact hen health and productivity. This scientific approach assesses crucial properties like bone strength, elasticity, and resistance to fractures, providing insights that are vital for improving hen welfare and egg production methods. By examining how bones respond under various conditions, researchers can identify specific risk factors and develop strategies to mitigate KBD.

The elastic modulus is an indicator that measures a material’s ability to resist elastic deformation. Some studies have shown that the elastic modulus of keel bones of normal and healthy laying hens may be within a certain range, but the specific value varies due to factors such as research methods, chicken breeds, and ages [108]. Research methodologies significantly influence these measurements. Variations in testing equipment, such as differences in precision, loading capabilities, and control mechanisms, can introduce inaccuracies if not properly calibrated [109]. Additionally, sample preparation procedures and environmental conditions during testing, including temperature and humidity, are crucial factors. Chicken breeds also contribute to the variability. Genetic differences among breeds impact bone structure and growth patterns [14], while differences in size and weight can lead to variations in bone density and, consequently, the elastic modulus. Age is another determinant [24]. Young hens experience developmental changes in their bones, with the elastic modulus evolving as they grow. In old age, a decline in the elastic modulus may occur due to bone degeneration processes, such as calcium loss and damage to the microstructure. 

#### Method for Biomechanical Determination of the Keel

Due to the complex geometry of the keel, including bending, twisting, uneven thickness and irregularities [110], it is impossible to calculate its material properties as existing models for regular shapes do not apply and stress/strain are unevenly distributed. In the determination of keel biomechanics, a mechanical testing apparatus (Stevens CR analyzer, Mechtric Engineering, Bristol, UK) was used [111]. The keel was placed on a custom support. Tests were conducted at the manubrial spine (position “A”) and the lateral surface (position “B”) of the keel. A 6.8 mm (or 7 mm) blunt probe was used to load the keel to failure at a constant rate of 50 mm/min [110,111]. The force (load) required to reach structural failure was recorded as a key indicator of the keel’s biomechanical properties. The results [110] show that from 30 weeks to 70 weeks of age, the bearing capacity of position A is about 25~40 N, and that of position B is about 12~20 N. At the same time, with the increase of age, the bearing capacity of the two locations showed irregular changes. Therefore, we speculated that the bearing capacity of laying hens with good bone development and healthy body may be greater in the peak laying period. However, for laying hens with keel damage, disease or malnutrition, the keel carrying capacity will be significantly reduced.

### 4.5. Behavioral Observations

The intricate relationship between behavioral changes and keel bone injuries in laying hens has been elucidated through meticulous observational studies. Typically, healthy hens display a range of active behaviors, such as walking, foraging, and stretching their wings. Conversely, hens afflicted with keel bone injuries often exhibit significant behavioral alterations; these include diminished mobility, evident lameness, and a tendency to isolate themselves, which are indicative of discomfort and can serve as preliminary signs of underlying skeletal issues [14]. Behavior is not a way to identify if a hen has keel damage. But it could be side evidence to identify the damage to the keel. We can make the final confirmation through behavioral observation, supplemented by X-ray machines and palpation.

Whereas healthy hens can perch with ease [20], injured hens experience difficulty due to pain and impaired balance, often forgoing higher perches for lower, more accessible resting places. This change not only affects their physical state but also their engagement in essential behaviors that contribute to their well-being.

Social interactions are equally affected by keel bone injuries. Under normal conditions, hens are socially active, engaging in grooming and interactive with peers [30]. However, hens with keel bone injuries show a marked decrease in these social behaviors, participating less frequently and becoming noticeably withdrawn [30]. This reduction in social interaction can lead to a decreased status within the flock’s hierarchy, impacting their access to resources like food and potentially altering their feeding patterns and social standing.

The effectiveness of interventions for keel bone injuries can be assessed by observing changes in these behaviors. Recovery is typically indicated by an improvement in mobility, an increase in social and perching activities, and a general return to normal behavior patterns [20]. If no improvement is observed, or if the hen’s condition deteriorates, this signals a need to reevaluate and possibly adjust the treatment strategies.

### 4.6. Biochemical Markers

In the evaluation of keel bone injuries in laying hens, various biological markers provide significant insights into the underlying physiological changes. Key among these are markers of inflammation and bone metabolism, which offer crucial data on the body’s response to injury and subsequent healing processes [2].

C-reactive protein (CRP) is an essential inflammation marker that increases in the blood following tissue damage or inflammation, indicative of keel bone injuries [112]. Similarly, interleukins such as IL-1 and IL-6, which play roles in the initiation and regulation of inflammatory responses, are elevated in cases of bone injury [24]. IL-1 is involved in activating the inflammatory cells at the onset of injury, while IL-6 helps in managing the immune response, thus indicating the extent of tissue reaction to the injury [24].

In terms of bone metabolism, alkaline phosphatase (ALP) and osteocalcin are critical indicators. ALP, an enzyme crucial for bone formation, shows increased activity as the body initiates bone remodeling to repair damaged areas [113]. Osteocalcin, a protein involved in bone mineralization, varies in response to bone injury and repair, reflecting the ongoing metabolic processes in bone tissue.

Collagen, a primary structural component of bone, also provides valuable markers [18]. Changes in collagen metabolism post-injury, indicated by levels of collagen breakdown products such as CTX-I, help assess the bone’s response to injury and its repair mechanisms. These changes are pivotal for understanding the dynamics of bone healing.

Additionally, oxidative stress markers like malondialdehyde (MDA) and superoxide dismutase (SOD) offer insights into the cellular stress responses following an injury. MDA, a byproduct of lipid peroxidation, increases with elevated oxidative stress, indicating cell membrane damage [114]. Conversely, SOD, an antioxidant enzyme, increases its activity as a protective response against oxidative damage, helping to maintain cellular integrity under stress [115].

Hormonal markers such as parathyroid hormone (PTH) and calcitonin also shift in response to bone injury [116]. PTH, which regulates calcium and phosphorus balance, may increase due to disrupted mineral metabolism. Calcitonin levels may fluctuate as the body modulates osteoclast activity and bone calcium content in the healing process.

These biomarkers, while indicative of keel bone injuries, are not exclusive to this condition and can be influenced by other physiological or pathological states. Therefore, a comprehensive evaluation using these markers, alongside clinical assessments and imaging techniques, is essential for accurate diagnosis and effective management of keel bone injuries in laying hens. This integrative approach enhances the understanding and treatment of such injuries, ultimately improving poultry welfare.

## 5. Management Strategies

To effectively mitigate KBD, a comprehensive management strategy that includes dietary adjustments, genetic selection, and environmental modifications is essential. This integrated approach aims to reduce both the prevalence and severity of KBD, ultimately enhancing the welfare and productivity of the flock.

### 5.1. Nutritional Strategies

Proper nutrition plays a crucial role in maintaining bone health, making dietary management a key component of KBD prevention. Calcium, phosphorus, and vitamin D3 are vital for strong bone development and maintenance [66,73,82]. An adequate and balanced intake of these nutrients helps ensure robust skeletal health. During peak laying periods, when the demand for these minerals is especially high due to egg production, supplementing the diet with additional calcium and vitamin D3 can help to reinforce bone strength, thereby making the keel bone less susceptible to fractures.

Furthermore, the incorporation of omega-3 fatty acids into the diet is beneficial [110]. These nutrients, found in flaxseed and fish oil, have anti-inflammatory properties that can aid in reducing the inflammation associated with bone injuries. This not only helps in managing pain but also supports overall bone health, potentially reducing the incidence of KBD.

### 5.2. Genetic Selection

Selective breeding represents another fundamental strategy for KBD prevention [117]. By choosing genetics that emphasize robust skeletal structures, poultry breeders can inherently decrease the birds’ predisposition to keel bone fractures [60]. This approach involves selecting for traits that enhance bone density and improve body conformation, which are crucial for reducing the risk of KBD. However, it is essential that this genetic selection does not compromise other desirable traits, such as egg production and temperament [60,117]. This balance ensures that while skeletal robustness is enhanced, the overall health and productivity of the birds are not adversely affected.

### 5.3. Environmental Modifications

The living environment plays a significant role in the incidence of KBD. Factors such as overcrowding and poor housing conditions can contribute to increased instances of collisions, which are common causes of keel bone injuries [11,55]. By providing adequate space per hen, the risk of stress and physical altercations, which can lead to injuries, is considerably reduced [15,52]. In addition to space management, the design of the housing environment is critical. The layout and accessibility of perches must be tailored to accommodate the hen’s anatomy to prevent falls and related injuries. Utilizing lower and wider perches can help in achieving this, as poorly designed perching systems are a notable risk factor for KBD [19,20].

### 5.4. Optimizing Lighting and Flooring

Adjusting lighting conditions within the henhouse can also play a crucial role in reducing stress and erratic behavior that may lead to injuries. A well-regulated lighting schedule can create a calmer atmosphere conducive to reducing behaviors that contribute to KBD. Proper lighting helps maintain a tranquil environment, which is essential for preventing hyperactivity that can cause accidents [118].

Additionally, the choice of flooring material is important in minimizing injury severity. Cushioned or rubberized flooring materials provide a softer landing for hens, absorbing the impact during falls and potentially preventing fractures.

### 5.5. Behavioral Enrichments

Providing environmental enrichments is another strategy to prevent KBD. These enrichments, which include dust baths, foraging substrates, and pecking blocks, encourage natural behaviors among hens. Engaging in these activities reduces boredom and stress, which are often precursors to aggressive interactions that can lead to physical harm [119].

## 6. Conclusions

In conclusion, the effective mitigation of KBD in laying hens necessitates concerted efforts across diverse domains, namely nutrition, genetics, environment, and animal behavior. Such comprehensive management not only ameliorates the welfare and prolongs the lifespan of the hens but also augments the overall efficiency and sustainability of poultry operations. Continued research and innovation are of utmost importance in devising more efficacious solutions and deepening the comprehension of KBD, thereby buttressing the ongoing endeavors to enhance the welfare standards and productivity within the egg-laying industry.

## Data Availability

The original contributions presented in this study are included in the article. Further inquiries can be directed to the corresponding authors.
